# Supplementary material for: FARSB Facilitates Hepatocellular Carcinoma Progression by Activating the mTORC1 Signaling Pathway
Source: Int J Mol Sci. 2023 Nov 24;24(23):16709. doi: 10.3390/ijms242316709 (PMC10706030; doi:10.3390/ijms242316709)
Supplement: Supplementary file 1 [file ijms-24-16709-s001.zip › ijms-2723191-supplementary.pdf]

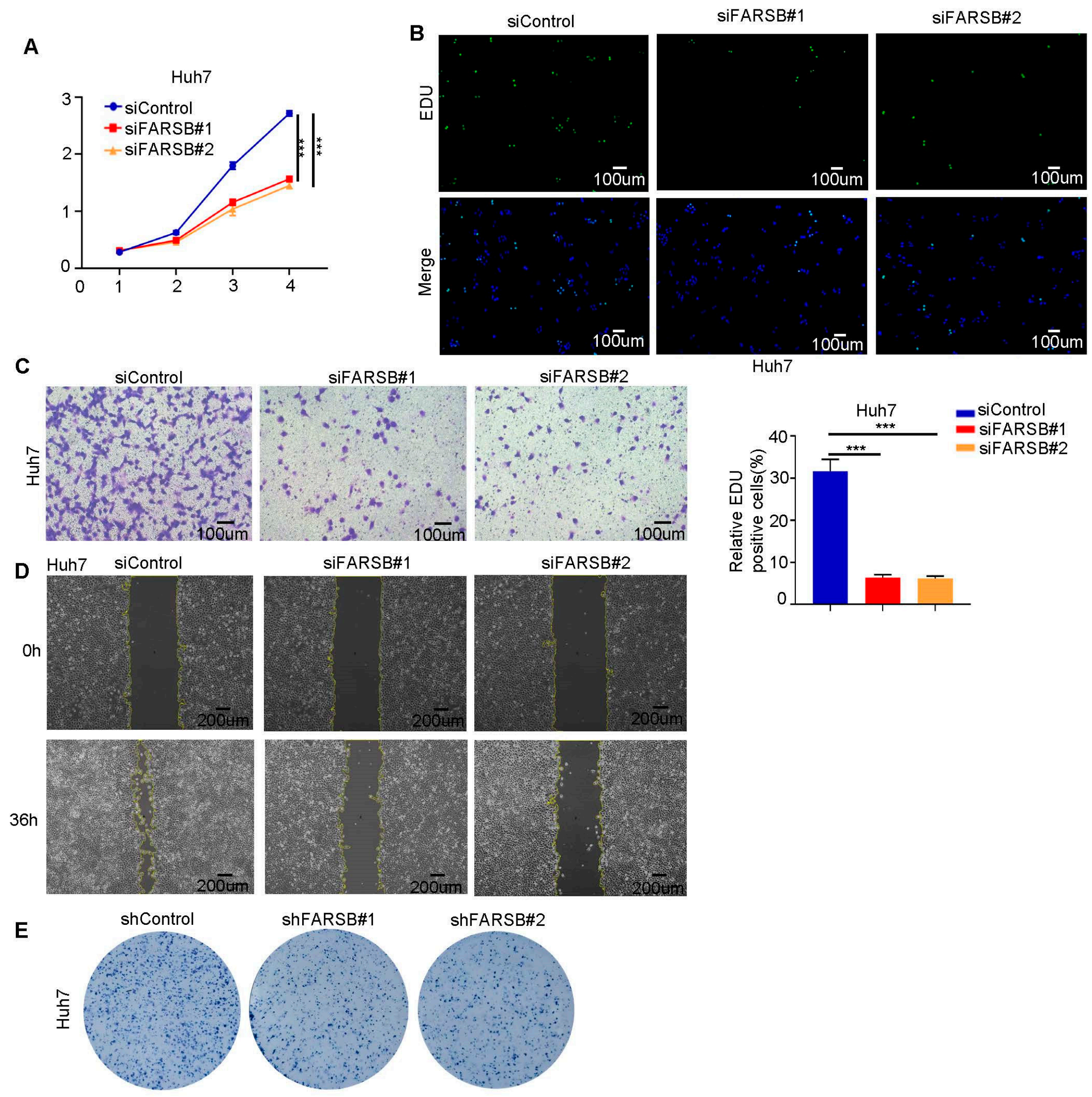

Figure S1. FARSB knockdown suppresses the proliferation and migration of Huh7 cells. (A-B) CCK-8 (A) and EDU (B) showed that knockdown of FARSB suppressed the proliferation of Huh7 cells, scale: 100μm. Fluorescent images showing EdU positive cells nuclei (green) and total nuclei in the samples (Hoechst, blue). (C-D) Transwell (C) and wound-healing assay (D) showed that knockdown of FARSB suppressed cell migration of Huh7 cells. Scale: 100μm. (E) Colony formation assay showed that the colony formation ability of FARSB knockdown Huh7 cells decreased. *** : $P < 0.001$.

A

	SYMBOL	RANK IN GENE LIST	RANK METRIC SCORE	RUNNING ES	CORE ENRICHMENT
1	EIF4E2	104	0.632	0.044	Yes
2	BRAF	1337	0.478	0.0576	Yes
3	CAB39	1634	0.461	0.0859	Yes
4	RPS6KA3	1943	0.447	0.1131	Yes
5	MAPK3	1981	0.445	0.1447	Yes
6	RPS6KB1	2085	0.44	0.1749	Yes
7	TSC1	2449	0.425	0.1995	Yes
8	MAPK1	2581	0.419	0.2276	Yes
9	EIF4B	2882	0.407	0.252	Yes
10	RHEB	2992	0.403	0.2793	Yes
11	MLST8	2998	0.403	0.3085	Yes
12	RICTOR	3039	0.401	0.3369	Yes
13	RPS6	3196	0.396	0.3629	Yes
14	STRADA	3517	0.384	0.3852	Yes
15	RPTOR	3535	0.383	0.4127	Yes
16	EIF4E	4446	0.357	0.4231	Yes
17	EIF4EBP1	4837	0.346	0.4414	Yes
18	HIF1A	5390	0.331	0.4559	Yes
19	PIK3R2	5615	0.325	0.4757	Yes
20	PIK3CA	5996	0.316	0.492	Yes

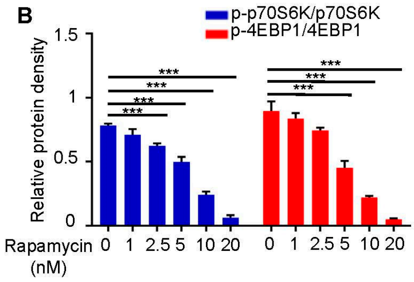

Figure S2. (A) Using the TCGA-LIHC data set, GSEA performed Kegg enrichment analysis, and the core molecules of the mTOR signaling pathway were significantly enriched in the FARSB high expression group. (B) The activation level of mTORC1 in MHCC97H cells treated with rapamycin at a gradient concentration for 24 h. *** : $P < 0.001$.

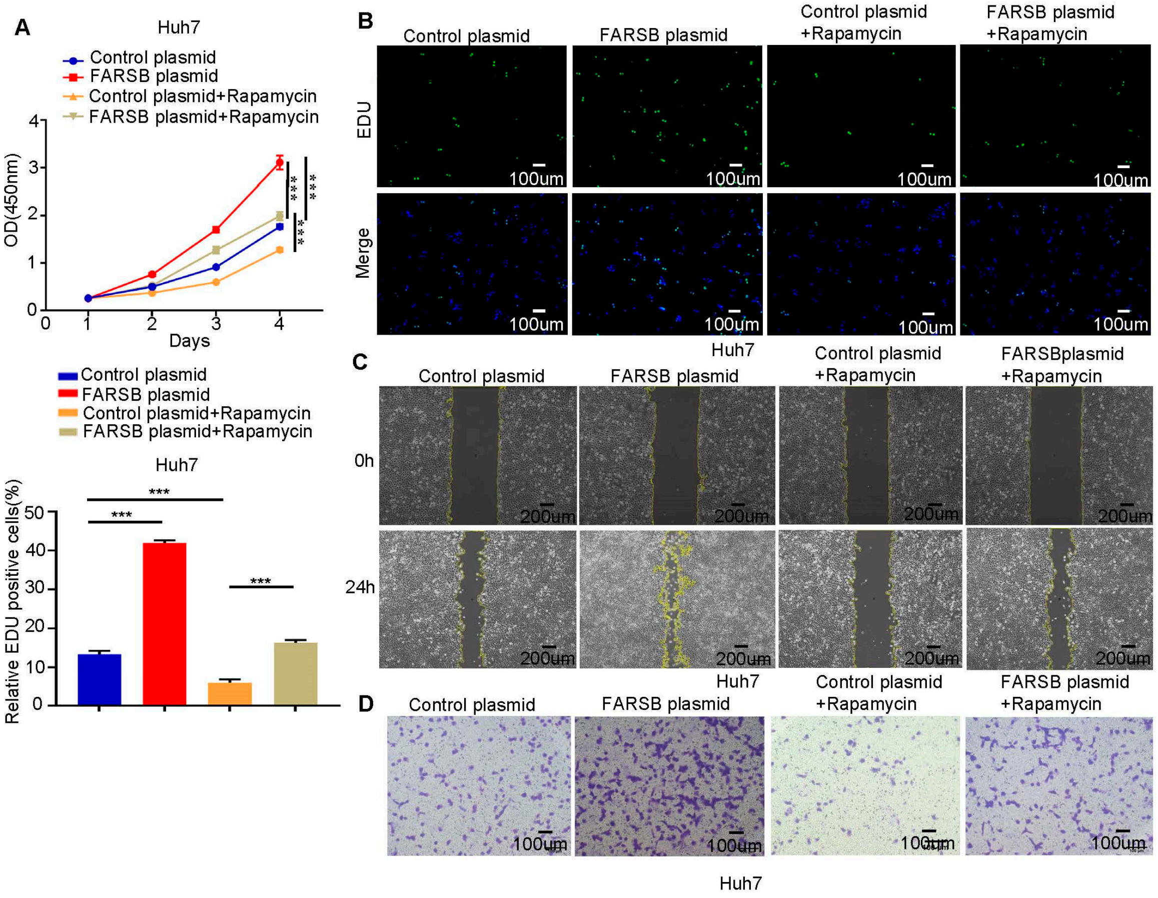

Figure S3. FARSB promotes the proliferation and migration of HCC cells by activating the mTORC1 signaling pathway. (A-D) Overexpression of FARSB and control group Huh7 cells were treated with 20 nM rapamycin for 48 h. CCK-8 (A) and EDU (B) were used to detect the proliferation of Huh7 cells. Fluorescent images showing EdU positive cells nuclei (green) and total nuclei in the samples (Hoechst, blue). Wound-healing assay (C) and Transwell assay (D) were used to detect the migration ability of Huh7 cells. *** : $P < 0.001$.

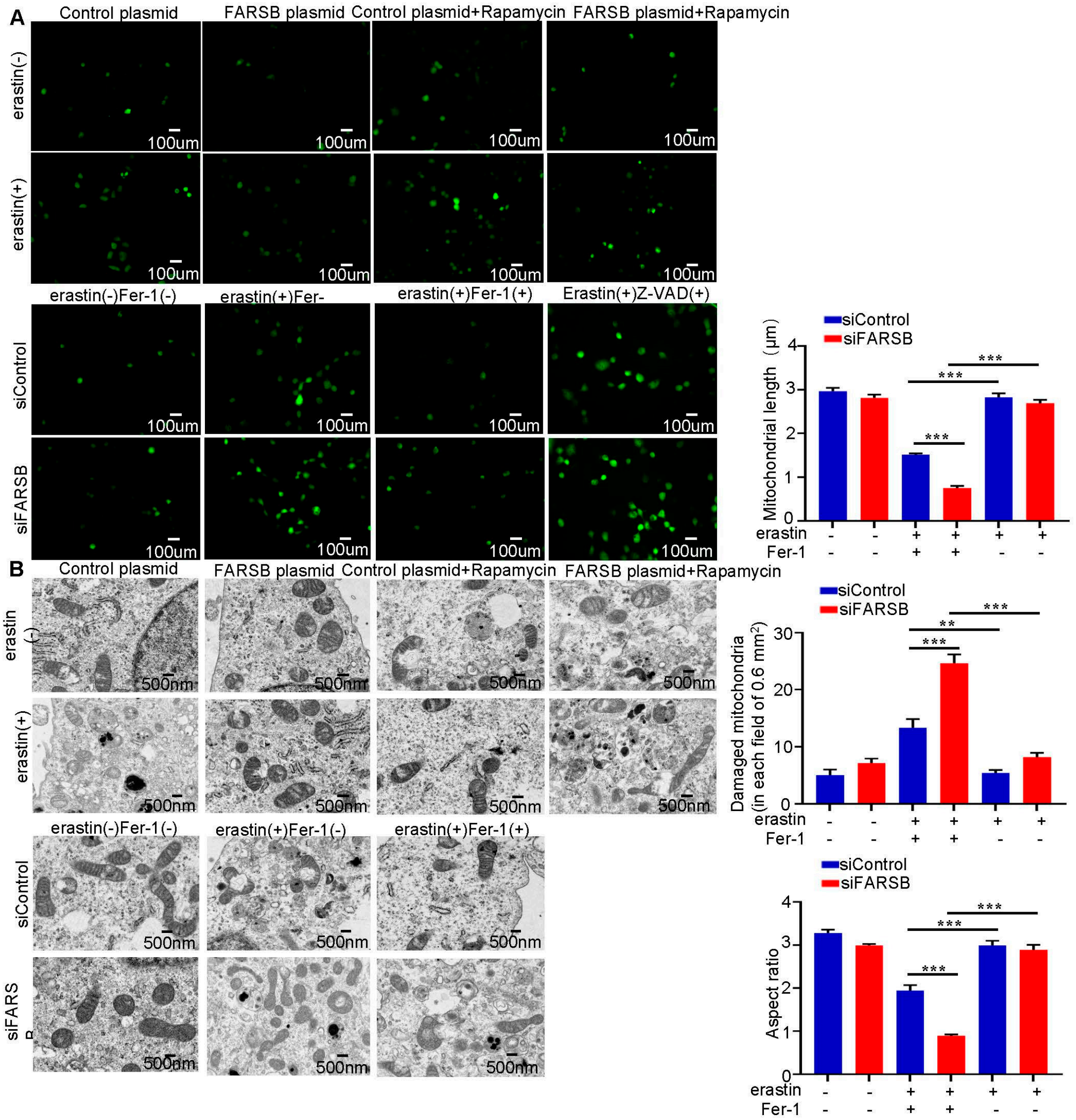

Figure S4. (A) MHCC97H cells with knockdown or overexpression of FARSB were treated with 10 μM erastin, and ROS was detected with or without ferrostatin-1 (2 μM), Z-VAD (10 μM) or rapamycin (20 nM). (B) (with or without 10μM erastin) Knockdown of FARSB (with or without 2μM ferrostatin-1), overexpression of FARSB (with or without 20nM rapamycin) and control group MHCC97H cells were observed under electron microscope. The morphology of mitochondria in each group was observed under the electron microscope, and the length of mitochondria, the number of mitochondrial damage and the aspect ratio were counted.** : $P < 0.01$, *** : $P < 0.001$.
