# Supplementary material for: FARSB Facilitates Hepatocellular Carcinoma Progression by Activating the mTORC1 Signaling Pathway
Source: Int J Mol Sci. 2023 Nov 24;24(23):16709. doi: 10.3390/ijms242316709 (PMC10706030; doi:10.3390/ijms242316709)
Supplement: Supplementary file 1 [file ijms-24-16709-s001.zip › sirna,shrna,primer.pdf]

Gene	Primer	Sequences
β-actin	Forward	5’- CCTGGCACCCAGCACAAT-3’
	Reverse	5’-GGGCCGGACTCGTCATAC-3’
FARSB	Forward	5’- GCCCTGTGCTCCCAAGAAGATA -3’
	Reverse	5’- AGGAAGAAGGGTAGTGCGTGCC -3’

Gene	siRNA	Sequences
SiFARSB#1	Forward	5’-GCAUGUGAUAUUGUAGAAGAU-3’
	Reverse	5’-AUCUUCUACAAUAUCACAUGC-3’
SiFARSB#2	Forward	5’- GCUGGACAGAAUUAUGCAGUU-3’
	Reverse	5’- AACUGCAUAAUUCUGUCCAGC-3’
siControl	Forward	5’- UUCUCCGAACGUGUCACGUTT-3’
	Reverse	5’- ACGUGACACGUUCGGAGAATT-3’

Gene	Sequences
shFARSB#1	5’-GCAUGUGAUAUUGUAGAAGAU-3’
shFARSB#2	5’- GCUGGACAGAAUUAUGCAGUU-3’
shControl	5’- UUCUCCGAACGUGUCACGUTT-3’
