# Supplementary material for: FARSB Facilitates Hepatocellular Carcinoma Progression by Activating the mTORC1 Signaling Pathway
Source: Int J Mol Sci. 2023 Nov 24;24(23):16709. doi: 10.3390/ijms242316709 (PMC10706030; doi:10.3390/ijms242316709)
Supplement: Supplementary file 1 [file ijms-24-16709-s001.zip › Supplementary material.pdf]

Table S1 Baseline characteristics of patients whose samples were used in the tissue microarray

**Clinical information**	**N(%)**
No. of patients	65
**Age ,yr**	**59.2±10.3**
**Gender (male/female)**	**42/21**
**Histology**	**Hepatocellular carcinoma**
**Stage**	
Ⅰ	5 (7.7%)
Ⅱ	15 (23.1%)
Ⅲ	33 (50.8%)
Ⅳ	12 (18.5%)
**T stage**	
T1	3 (4.6%)
T2	13 (20%)
T3	38 (58.5%)
T4	11 (16.9%)
**Lymph nodes**	
N0	20 (30.8%)
N1	12 (18.5%)
N2	18 (27.7%)
N3	15 (23.1%)
**Metastasis**	
M0	52 (80%)
M1	13 ((20%)
**Grade**	
G2	13 (20%)
G2-3	14 (21.5%)
G3	31 (47.7%)
G3-4	7 (10.8%)
